# Supplementary material for: Quantitative Evaluation of a Fully Automated Planning Solution for Prostate-Only and Whole-Pelvic Radiotherapy
Source: Cancers (Basel). 2024 Nov 5;16(22):3735. doi: 10.3390/cancers16223735 (PMC11591666; doi:10.3390/cancers16223735)
Supplement: Supplementary file 1 [file cancers-16-03735-s001.zip › Supplementary Figures S1-S3.pdf]

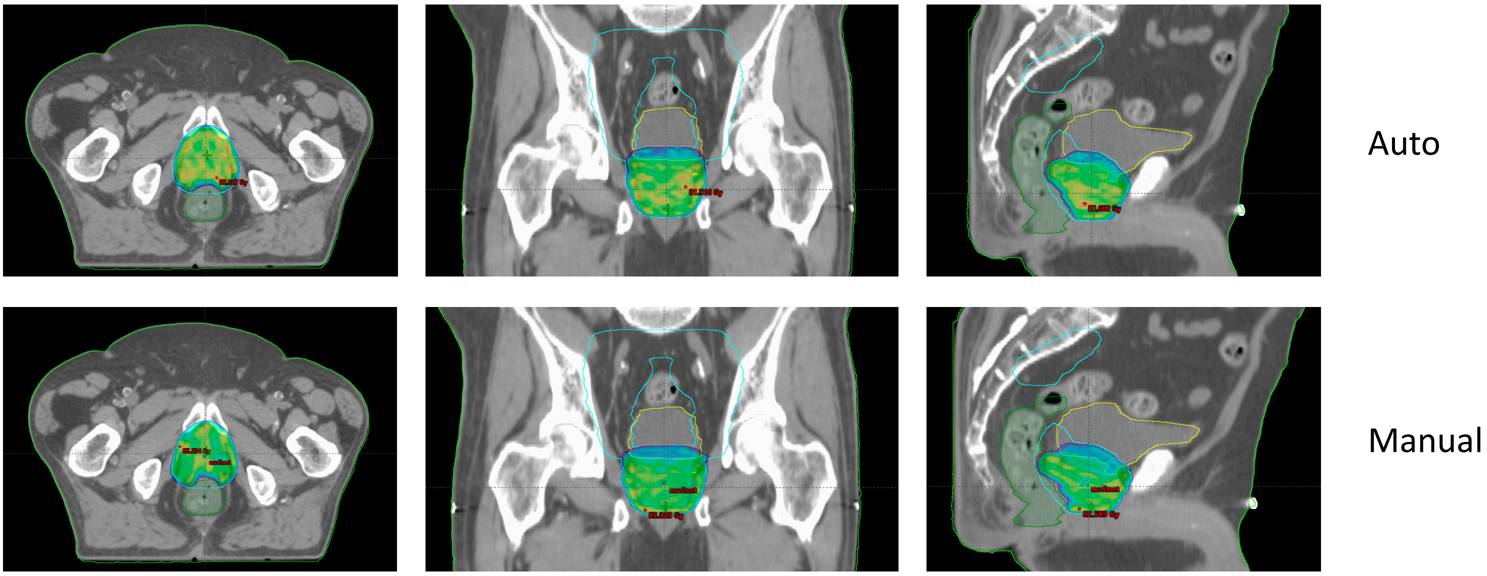

Figure S1. Example of 76Gy isodose for automated (upper) and manual treatment plans for whole-pelvic localization (Patient n° 5).

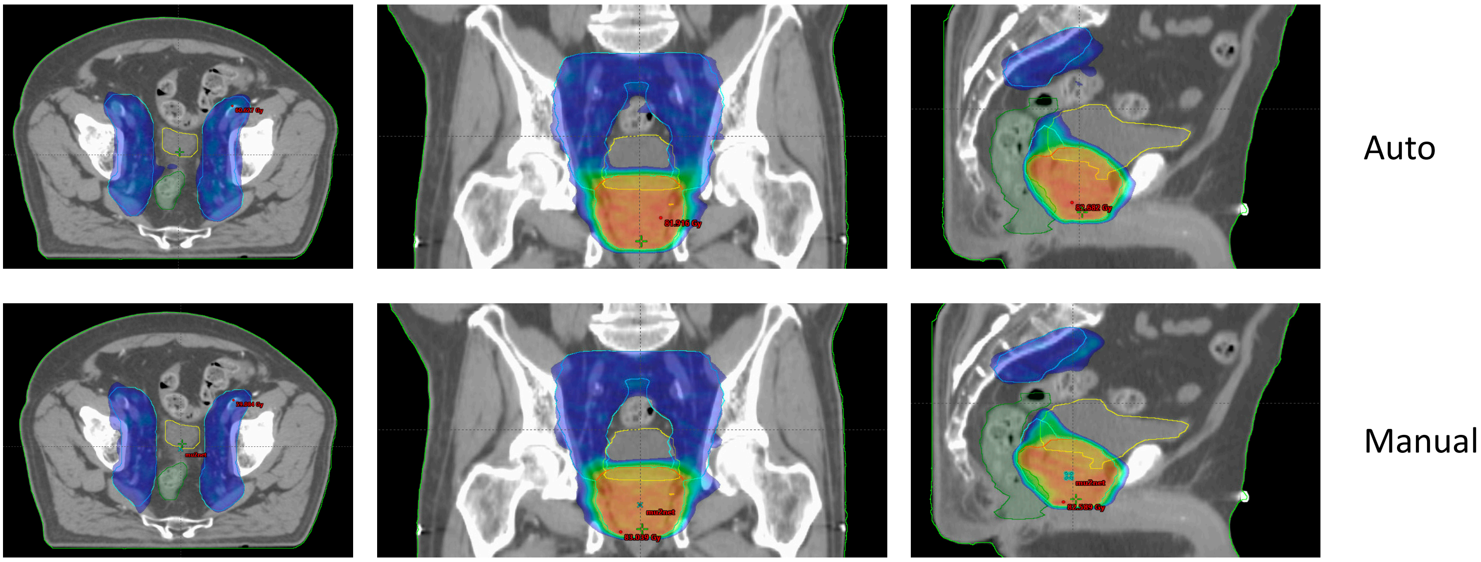

Figure S2. Example of 53.2Gy isodose for automated (upper) and manual (lower) treatment plans for whole-pelvic localization (Patient n° 5).

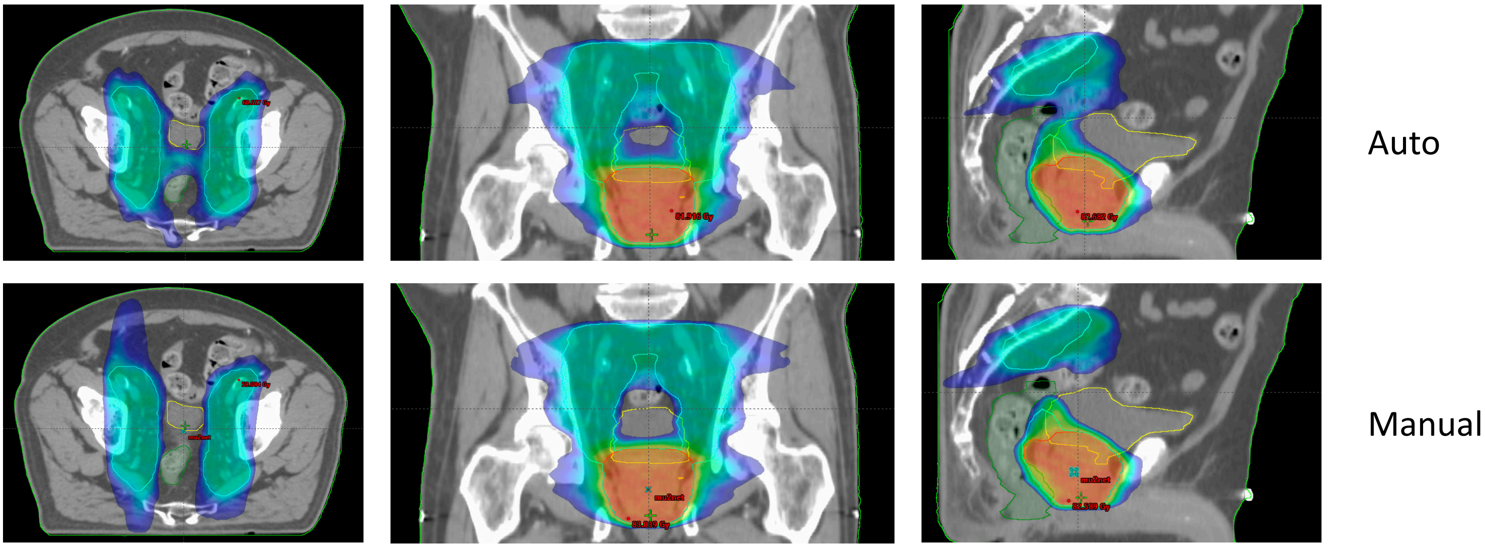

Figure S3. Example of 40Gy isodose for automated (upper) and manual (lower) treatment plans for whole-pelvic localization (Patient n° 5).
