# Supplementary material for: Quantitative Evaluation of a Fully Automated Planning Solution for Prostate-Only and Whole-Pelvic Radiotherapy
Source: Cancers (Basel). 2024 Nov 5;16(22):3735. doi: 10.3390/cancers16223735 (PMC11591666; doi:10.3390/cancers16223735)
Supplement: Supplementary file 1 [file cancers-16-03735-s001.zip › Supplementary Table S1.pdf]

Table S1. Details of patient cohort including PTVs volumes, treatment machine and VMAT geometry.

		Volume (cc)		Linac	VMAT geometry	
	Patient n°	PTV2	PTV1		Number of arcs	Collimator angles (°)
Prostate-only	1	118,07	178,77	TrueBeam STx	2	45/315
	2	119,1	160,1		1	45
	3	217,82	296,8		1	45
	4	114,37	140,45		1	30
	5	127,72	195,44		1	30
	6	201,59	254,12		1	45
	7	97,86	128,7		2	45/315
	8	152,62	180,43		1	45
	9	156,36	208,62		1	45
	10	147,95	188,79		1	45
	11	161,88	247,94		1	45
	12	134,44	176,88		1	45
	13	117,78	132,04		2	45/315
	14	167,66	211		2	30/330
	15	90,48	133,31		1	45
	16	128,01	175,33		1	45
	17	154,97	219,47		2	45/315
	18	221,42	283,07		2	45/315
	19	108,45	175,73		2	45/315
	20	88,01	139,45		1	45
Whole-pelvic	1	146,2	776,25	Ethos	4	281/326/11/56
	2	198,23	831,39	Ethos	4	281/326/11/56
	3	182,6	1051,91	Ethos	3	345/15/345
	4	139,08	959,82	TrueBeam STx	2	30/330
	5	141,43	807,97	TrueBeam STx	2	30/330
	6	135,46	630,39	TrueBeam STx	2	20/340
	7	187,57	1032,36	Ethos	4	281/326/11/56
	8	104,52	864,23	TrueBeam STx	2	20/340
	9	126,04	631,2	TrueBeam STx	2	20/340
	10	303	1156,67	TrueBeam STx	2	30/330
	11	144,92	904,81	Ethos	4	281/326/11/56
	12	88,48	819,2	TrueBeam STx	2	45/315
	13	109,32	817,62	TrueBeam STx	2	30/330
	14	112,94	861,23	TrueBeam STx	2	30/330
	15	104,33	797,49	TrueBeam STx	2	45/315
	16	100,35	779,38	TrueBeam STx	2	30/330
	17	97,82	846,96	TrueBeam STx	2	20/340
	18	141,17	746,05	TrueBeam STx	2	30/330
	19	80,8	1268,27	TrueBeam STx	2	30/330
	20	103,22	793,83	TrueBeam STx	2	30/330
